# Validation of the French versions of the Hirschsprung’s disease and Anorectal malformations Quality of Life (HAQL) questionnaires for adolescents and adults

**DOI:** 10.1186/s12955-017-0599-7

**Published:** 2017-01-28

**Authors:** Corine Baayen, Fanny Feuillet, Pauline Clermidi, Célia Crétolle, Sabine Sarnacki, Guillaume Podevin, Jean-Benoit Hardouin

**Affiliations:** 1 UMR 1246 INSERM SPHERE “methodS in Patient-centered outcomes & HEalth ResEarch”, University of Nantes, Nantes, France; 2 Plateforme de Biométrie, Département Promotion de la Recherche Clinique, University Hospital, Nantes, France; 3 Department of Pediatric surgery, Robert Debré Hospital, Paris, France; 4 National French Center of Expertise for Anorectal and Rare Pelvic Malformations, Paris, France; 5 Pediatric Surgery and Urology Department, AP-HP, Necker Hospital, Paris, France; 6 University Paris Descartes, Sorbonne Paris Cite, Paris, France; 7 Department of Pediatric Surgery, University Hospital, Angers, France

**Keywords:** Anorectal malformation, Cultural adaptation, Disease-specific questionnaire, HAQL, Hirschsprung’s disease, Krickenbeck, Quality of Life

## Abstract

**Background:**

The Hirschsprung’s disease Anorectal malformation QoL questionnaire (HAQL) is a disease-specific quality of life (QoL) questionnaire for patients with Hirschsprung’s disease (HD) or anorectal malformations (ARM). It was originally proposed in Dutch and is currently being translated into other languages to obtain an internationally standardized instrument. In this work we validate a French adaptation of the HAQL for adolescents and adults.

**Methods:**

The questionnaires were translated into French and sent to patients aged 12 years and older, followed for HD or ARM at three French university hospitals. Questionnaires were sent to 147 adolescents and 188 adults. The psychometric properties of the questionnaires were analyzed in terms of reliability and validity.

**Results:**

The original HAQL structure was not satisfactory. A new structure was proposed, while aiming to remain close to the original structure. The proposed structure has acceptable reliability and validity properties and reflects both physical, as well as psychosocial aspects.

**Conclusions:**

A French version of the HAQL questionnaire for adults and adolescents is ready for use in France. In particular the score could discriminate between degrees of clinical status based on the Krickenbeck consensus, which can aid clinicians to inform patients about physical and psychosocial challenges they may expect.

**Electronic supplementary material:**

The online version of this article (doi:10.1186/s12955-017-0599-7) contains supplementary material, which is available to authorized users.

## Background

Hirschsprung’s disease (HD) and anorectal malformations (ARM) are rare congenital defects which occur during fetal development and which can have life-long consequences. Both conditions require neonatal surgery and some patients may need a colostomy (stoma). However, even a very successful anatomic repair may not be able to ensure optimal bowel control, urinary control, or sexual functioning. For patients with lasting functional digestive symptoms, regular follow-up is required and bowel management programs involving enemas and dietary restrictions have been developed to improve their quality of life (QoL) [1].

QoL evaluation tools are useful to aid further treatment decisions for such patients, as well as to support a reassessment of their prognosis. They are also useful to facilitate further research on the QoL of these patients, for example to identify subgroups of patients with a high risk of a low QoL and to understand how any QoL related problems can be prevented or managed.

Whereas general QoL questionnaires are available, disease specific measures provide greater sensitivity and specificity. Therefore, in 2001, Hanneman and colleagues constructed the Hirschsprung’s disease Anorectal malformation QoL questionnaire (HAQL), a self-report instrument to assess QoL in patients with HD or ARM [2]. They proposed different versions of the questionnaire depending on the age of the patient: a proxy form for children of 6, or 7 years old, a proxy and a patient form for children of 8 to 11 years old and adolescents of 12 to 16 years old and a patient form for adults of 17 years and older.

The HAQL was originally formulated in Dutch. To ensure an internationally standardized evaluation tool, the questionnaires are being translated and culturally adapted to other languages such as Italian and Swedish [3, 4]. The child version for children of 6 to 11 years old has been translated to French and was validated in a study by Clermidi and colleagues [5]. The aim of the present study is to validate a French translation of the child and proxy questionnaires for patients from 12 to 16 years old (HAQL_12–16_ and HAQL_proxy_) and the adult form for patients of 17 years and older (HAQL_17+_).

## Methods

### Original HAQL questionnaires

The original HAQL questionnaires cover physical, emotional and social functioning, as well as disease-related symptoms [2]. The HAQL_12–16_ is composed of nine dimensions (40 items) [2, 6]: laxative diet (two items), constipating diet (two items), presence of diarrhea (two items), fecal continence (eight items), urinary continence (four items), social functioning (five items), emotional functioning (six items), body image (two items) and physical symptoms (nine items). For the adults, the questionnaire is composed of ten dimensions (41 items): the nine same dimensions as the HAQL_12–16_ (with small differences in the number of items in the social functioning (three items) and the emotional functioning (seven items) dimensions) and a sexual functioning dimension (two items).

For each item the response is scored from 0 to 3 and linearly transformed to a 0 to 100 scale, so that higher scores indicate a better QoL. The scores for the dimensions are computed as the sum of the item scores, divided by the number of items answered, conditional on that more than 50% of the items are answered. If information on all dimensions is available for a patient, a total score can be computed as the sum of the scores of the dimensions.

For patients with a stoma, an extra section is included related to the stoma (eight items). These patients are asked to skip part of the questions (22 items) for patients without a stoma, since these questions are not relevant for them, e.g. questions related to going to the toilet.

The questionnaires were translated into French using standard methodology [7–9].

### Study design

All patients followed for HD or ARM at the University Hospitals of Nantes, Angers and Paris-Necker (French center of expertise for anorectal and rare pelvic malformations) were contacted if they were aged 12 or older in April 2011. In accordance with the exclusion criteria used by Hanneman and colleagues [2], patients were excluded if they had a cloaca, were mentally retarded, or lacked a basic proficiency in French.

Patients received a mail with the appropriate HAQL questionnaires and a generic questionnaire on QoL: the Vécu et Santé Perçue de l’enfant et de l’Adolescent questionnaire (VSPA) [10] for adolescents and the short version of the World Health Organization Quality Of Life questionnaire (WHOQOL BREF) [11] for adults. In addition, they received an information letter, a face validity questionnaire and a questionnaire containing the Krickenbeck criteria [12] to measure clinical status. After two weeks, all patients received a conditional reminder asking them to return the questionnaire if they hadn’t done so yet. Four weeks after the initial mail, all patients received another mail including only an information letter and the HAQL and clinical status questionnaires (retest step).

The research protocol has been validated by an ethical committee: the Groupement Nantais d’Ethique dans le Domaine de la Santé (GNEDS) which is specialized in non-interventional studies. The protocol is registered under the reference 2011-06-01.

### Evaluation of the French version of the HAQL

All statistical analyses were performed in SAS® (version 9.3, SAS Institute Inc.) and R version 3.2.2. Data are expressed by mean ± standard deviation. The significance level was fixed at 0.05. All correlations are expressed in Pearson’s correlation coefficient (denoted by ρ).

#### Validity

The validity of the French translation of the HAQL was assessed in terms of face validity, concurrent validity and construct validity. The content validity of the HAQL was considered by Hanneman et al. [2] for the Dutch versions of the questionnaires. It was not re-evaluated for the French questionnaires, since cultural differences between the Dutch and French populations are minor, in particular concerning questionnaires based on the impact of symptoms on Health related QoL. Experts at the three French University Hospitals did not underline any problems concerning the content validity.

Face validity verifies whether the questionnaire appears relevant and comprehensible to the participants. It was assessed through a small questionnaire sent to all patients in the study, which evaluated response time and whether there were any problems to understand the questions.

Construct validity indicates whether the structure of the questionnaire (the relations between items and dimensions) is valid. It was studied using a multitrait-scaling analysis (MTS) and known-groups validity. The MTS analysis in particular assessed convergent and divergent validity, considering that each item should be highly correlated with its own dimension (ρ > 0.40), and should be more correlated with its own dimension than with others.

Concurrent validity compares an instrument with an established measure of the same, or a closely related construct. It was assessed by considering the correlations between the dimensions of the French HAQL and the generic QoL measures VSPA and WHOQOL BREF. The concurrent validity was considered satisfactory if the correlation between two close dimensions was higher than 0.40.

Known-groups validity evaluates whether an instrument can discriminate between known groups of patients that are expected to score differently on the measure of interest. It was assessed by comparing the mean scores of known groups of patients for each HAQL dimension using an ANOVA in case of multiple groups or Student’s *t*-test when two groups were compared. The groups were “disease status” (HD versus ARM) and groups based on clinical status according to the Krickenbeck classification (degrees of autonomy status, soiling, constipation and voluntary bowel movement).

#### Reliability

The reliability of the questionnaires was evaluated in terms of internal consistency and test-retest reliability. Internal consistency assesses whether a questionnaire measures a homogeneous concept by evaluating the correlations between items. For each dimension, it was measured by Cronbach’s α, where α > 0.70 was considered satisfactory [13].

Test-retest reliability evaluates the reproducibility of a questionnaire, i.e. whether a patient who is tested repeatedly in a stable condition reports similar results. The Intraclass Correlation Coefficient (ICC) was used to measure the agreement between the two sets of HAQL scores obtained from the patients at the start of the study and 4 weeks later. An ICC > 0.60 was considered satisfactory [13].

Finally, for adolescents, the concordance between the patient and proxy outcomes was assessed based on the ICC.

#### Methodology to propose a new structure

If validity of the original HAQL structure was not satisfactory, a new structure would be proposed based on the MTS analysis and expert opinions. To ensure comparability across countries, an effort would be made to keep the structure as close as possible to the original structure proposed by Hanneman et al. [2]. The validity and reliability of the new structure would be assessed using the same methodology as described above.

## Results

### Response rates

**Fig. 1:**
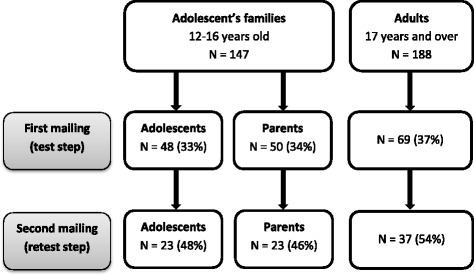
Flow-chart of patient inclusion in the study

One hundred forty-seven adolescent and proxy questionnaires and 188 adult questionnaires were sent out. The response rate for the first mailing (test step) was 33% (*n* = 48) for the adolescents, 34% (*n* = 50) for their proxies and 37% (*n* = 69) for the adults. The response rate for the second mailing (retest step) for the patients that had returned a questionnaire in the first step was 48% (*n* = 23) for the adolescents, 46% (*n* = 23) for their proxies and 54% (*n* = 37) for the adults (see Fig. 1).

The percentage of HD patients was 12% (*n* = 8) for adults and 33% (*n* = 16) for adolescents. Ten adults (14%) and three adolescents (6%) reported having a stoma. Five other adolescents indicated that they did not have a stoma, whereas their proxies indicated that they did. Since priority should be given to the patient’s answers [14] and since the questions for patients without a stoma can be answered in a sensible way, even when patients have a stoma, the answers of these patients to these questions were included in the analysis, with the exception of two patients who did not understand the term stoma.

### Dropout analysis

Patients which did or did not respond to the re-test step were compared based on their responses for the test-step. For each dimension and total score, a *t*-test was performed to evaluate whether scores of responders and non-responders differed. No significant differences were found, although total scores were slightly higher for non-responders (adolescents: 592 vs 623, *p*-value = 0.302, adults: 586 vs 652, *p*-value = 0.082). We therefore expect that there is no relevant difference between these patients and expect similar test-retest concordance results for patients with and without re-test responses.

We do not have sufficient information on patients which did not respond to the test step to compare them to the patients which did respond. Based on the above comparison, we may expect that on average non-responders are slightly less severe cases, although differences are likely to be small. However, the sample includes patients with extreme scores on the HAQL questionnaire, which indicates that the sample covers patients with a wide range of disease severity. For the purpose of validation of the questionnaire, we therefore do not expect a bias in the results.

### Face validity

The overall missing item response rate for the test step was 4.8% for the adolescents, 4.0% for their proxies and 1.5% for adults. For the retest step, these rates were 0.1, 0.5 and 1.2% respectively. The items related to sexual functioning had somewhat higher nonresponse rates than the other items (around 10%). No other specific item had an unusually high nonresponse rate. The average response times were 23.1, 25.6 and 19.8 min for adolescents, proxies and adults respectively.

Seven adolescents (15%) and 11 adults (16%) reported that the questions regarding urinary function were not relevant. Moreover, three adolescents (6%) and 11 adults (16%) mentioned that they considered the questions regarding a stoma either difficult or irrelevant. Two of these adolescents had a stoma according to their parents, but did not report having a stoma themselves. The remaining adolescent and one of the adults reported having a stoma, but considered the questions irrelevant. Finally, seven adults (10%) reported that they found the questions about their sexual functioning either intrusive or irrelevant.

### Validity of the original structure

Because of the small number of patients with a stoma, the questions concerning the stoma were not validated in this paper. For the remaining items, Additional file 1: Tables S1 and S2 in the appendix present the MTS analyses of the French translation of the HAQL questionnaires for adults, respectively adolescents. For the adults, out of 41 items, three items have poor convergent validity and 11 do not have satisfactory divergent validity. In particular, Dimension 8 “Body Image” performs poorly, since neither item satisfies divergent validity. For Dimension 2 “Constipating diet”, one out of its two items does not reach the validity criteria. For the adolescents, out of 40 items, five items have poor convergent validity and 24 do not reach divergent validity. Dimensions 2, 6 and 8 corresponding to “Constipating diet”, “Social functioning” and “Body Image” do not contain any items with satisfactory validity properties.

The above validity results for the original HAQL structure were considered unsatisfactory. Therefore, it requires improvement before it can be used in France.

### Proposition of a new structure

It was briefly assessed whether the HAQL structure proposed by Clermidi et al. [5] for children between 6 and 11 years old was suitable for adolescents and adults. For adults and adolescents respectively, MTS analyses revealed that five, respectively six out of 25 items did not meet convergent validity and ten, respectively 13 items did not meet divergent validity. Since these results are not better than the results for the original structure and since it seems best for international comparability to remain as close as possible to the structure used in other countries, it was decided to propose a new structure based on the original HAQL, but with several modifications based on the MTS results and expert opinions:

- Dimensions 2 “Constipating diet” and 3 “Presence of diarrhea” were grouped into one dimension, since they are closely related and for adolescents, the items in Dimension 2 correlated more strongly with Dimension 3 than with their own dimension. For adults Dimension 2 did not have satisfactory validity properties either.
- Dimensions 7 “Emotional functioning” and 8 “Body image” were grouped into one dimension, since the items in Dimension 8 correlated more strongly with Dimension 7 than with Dimension 8 for both adults and adolescents and the dimensions are close.
- Items “loss of feces during emotional moments” and “going to the toilet while having no urge” corresponding to item numbers 43 and 31 respectively for adolescents and 46 and 34 for adults, were removed. Also, items “amorous feelings” and “fantasize about making love” corresponding to item numbers 17 and 18 for adolescents were removed. These items had especially bad divergent as well as convergent validity properties and there was no clear indication that they could be moved to another dimension.
- Item 9 “important to be in neighborhood of a toilet” was moved from dimension “Fecal continence” to “Emotional functioning” for both adults and adolescents. This decision was based on that Item 9 correlated strongly with Dimension “Emotional functioning” for both age groups, while it reached neither convergent, nor divergent validity in its former dimension.

The resulting proposed structure for adolescents contains 36 items in 7 dimensions: Laxative diet, Presence of diarrhea, Fecal continence, Urinary continence, Social functioning, Emotional functioning and body image and Physical symptoms. For adults it encompasses a total of 39 items, with 37 items in the same 7 dimensions, plus one extra dimension “Sexual functioning” containing two items (Additional file 1: Tables S3 and S4). Descriptive statistics for the new scale can be found in Table 1.

**Table 1:** Description and reliability of the HAQL proposed structures for adults and adolescents

HAQL dimensions	N	% missing	Min	Max	Mean	Sd	Internal consistency Cronbach’s α coefficient	Test-retest concordance Intraclass correlation	Patient-proxy concordance Intraclass correlation
Overall scale	51	26%	309	800	620.86	135.29	0.95	0.91	–
Adults									
1 - Laxative Diet	68	1%	17	100	85.50	24.09	0.78	0.66	–
2 - Presence of Diarrhea	59	14%	0	100	77.69	22.85	0.76	0.90	–
3 - Fecal Continence	58	16%	6	100	82.88	21.83	0.88	0.86	–
4 - Urinary Continence	69	0%	0	100	91.17	18.79	0.91	0.75	–
5 - Social Functioning	59	14%	0	100	78.20	30.42	0.89	0.86	–
6 - Emotional Functioning and Body Image	69	0%	3	100	64.01	28.00	0.93	0.88	–
7 - Physical symptoms	59	14%	0	100	60.85	23.77	0.86	0.83	–
8 - Sexual functioning	61	12%	0	100	81.18	29.52	0.94	0.78	–
Adolescents									
Overall scale	42	12%	276	700	608.07	95.50	0.94	0.91	0.94
1 - Laxative Diet	48	0%	33	100	88.83	18.01	0.65	0.88	0.73
2 - Presence of Diarrhea	43	10%	17	100	83.35	25.09	0.88	0.73	0.92
3 - Fecal Continence	43	10%	28	100	85.70	18.92	0.85	0.92	0.86
4 - Urinary Continence	48	0%	58	100	97.60	7.29	0.78	0.93	0.71
5 - Social Functioning	42	12%	0	100	93.14	19.92	0.95	0.88	0.81
6 - Emotional Functioning and Body Image	48	0%	19	100	84.17	20.69	0.91	0.91	0.82
7 - Physical symptoms	43	10%	17	100	71.77	18.67	0.80	0.79	0.74

#### Validity

Concurrent validity results for the proposed structure can be found in Additional file 1: Tables S5 (adults) and S6 (adolescents). For adults, all dimensions reached a significant correlation > 0.40 with at least one close dimension from WHOQOL-BREF, except laxative diet (significant, but low correlation) and urinary continence. For adolescents, all HAQL dimensions significantly correlated with a coefficient > 0.40 to a relevant dimension from VSPA. The dimensions “Relations with family”, “School work” and “Relations with medical staff” from VSPA did not correlate significantly with any HAQL dimension.

The MTS analysis indicated that all items reached convergent validity for adults and all items, except Items 36 and 48, reached divergent validity (Additional file 1: Table S7). For adolescents, all items satisfied convergent validity, except Item 7 (*ρ* = 0.38). Divergent validity was satisfactory for all but ten items (Additional file 1: Table S8).

Results on known-groups validity can be found in Tables 2 (adults) and 3 (adolescents). Neither the overall score, nor any of the dimensions were able to discriminate between the disease statuses HD or ARM. For adults, the overall score was not able to detect different autonomy statuses (*p* = 0.2700), but could discriminate between different degrees of all other clinical status variables. All dimensions could discriminate between groups of at least one variable, except Dimension 4 “Urinary continence”. For each clinical status variable, there were at least three dimensions which could distinguish the different groups.

**Table 2:** HAQL Adults - Known-groups validity for the proposed score (significant results are given in boldface)

HAQL dimensions	Disease status^a^ HD *N* = 8 ARM *N* = 60	Clinical status			
		Autonomy status^a^ Usual A. *N* = 56 Lower A. *N* = 9	Soiling^b^ Grade 0 *N* = 33 Grade 1 *N* = 24 Grade 2 *N* = 4 Grade 3 *N* = 3	Constipation^b^ Grade 0 *N* = 36 Grade 1 *N* = 7 Grade 2 *N* = 6 Grade 3 *N* = 7	Voluntary bowel movements^b^ Grade 0 *N* = 30 Grade 1 *N* = 18 Grade 2 *N* = 9
Overall scale	621 ± 135 *p* = 0.55	621 ± 135 *p* = 0.27	**Grade 0: 706 ± 80** **Grade 1: 594 ± 129** **Grade 2 or 3: 473 ± 112** ***p*** **< 0.0001**	**Grade 0 or 1 or 2: 639 ± 135** **Grade 3: 462 ± 136** ***p*** **= 0.0121**	**Grade 0: 691 ± 103** **Grade 1 or 2: 544 ± 142** ***p*** **= 0.0005**
1- Laxative Diet	86 ± 24 *p* = 0.32	**Usual A.: 83 ± 26** **Lower A.: 98 ± 6** ***p*** **= 0.0004**	86 ± 24 *p* = 0.12	**Grade 0: 92 ± 18** **Grade 1 or 2 or 3: 69 ± 30** ***p*** **= 0.0025**	86 ± 24 *p* = 0.69
2- Presence of Diarrhea	78 ± 23 *p* = 0.34	78 ± 23 *p* = 0.85	**Grade 0 : 88 ± 16** **Grade 1: 76 ± 21** **Grade 2 or 3: 57 ± 31** ***p*** **= 0.0021**	78 ± 23 *p* = 0.80	**Grade 0: 90 ± 13** **Grade 1: 73 ± 17** **Grade 2: 35 ± 29** ***p*** **< 0.0001**
3- Fecal Continence	83 ± 22 *p* = 0.15	83 ± 22 *p* = 0.81	**Grade 0: 97 ± 7** **Grade 1: 82 ± 16** **Grade 2 or 3: 47 ± 27** ***p*** **< 0.0001**	83 ± 22 *p* = 0.57	**Grade 0: 94 ± 12** **Grade 1 or 2: 72 ± 21** ***p*** **< 0.0001**
4- Urinary Continence	91 ± 19 *p* = 0.93	91 ± 19 *p* = 0.19	91 ± 19 *p* = 0.24	91 ± 19 *p* = 0.15	91 ± 19 *p* = 0.31
5- Social Functioning	78 ± 30 *p* = 0.83	78 ± 30 *p* = 0.11	**Grade 0 or 1: 85 ± 25** **Grade 2 or 3: 29 ± 25** ***p*** **< 0.0001**	78 ± 30 *p* = 0.06	**Grade 0: 91 ± 15** **Grade 1 or 2: 62 ± 38** ***p*** **= 0.0028**
6- Emotional Functioning and Body Image	64 ± 28 *p* = 0.50	**Usual A.: 68 ± 26** **Lower A.: 39 ± 27** ***p*** **= 0.0128**	**Grade 0: 78 ± 22** **Grade 1: 58 ± 26** **Grade 2 or 3: 25 ± 17** ***p*** **< 0.0001**	64 ± 28 *p* = 0.12	**Grade 0: 78 ± 21** **Grade 1 or 2: 54 ± 29** ***p*** **= 0.0048**
7- Physical symptoms	61 ± 24 *p* = 0.21	**Usual A.: 64 ± 24** **Lower A.: 47 ± 19** ***p*** **= 0.0441**	**Grade 0 : 72 ± 20** **Grade 1: 56 ± 23** **Grade 2 or 3: 33 ± 20** ***p*** **= 0.0001**	**Grade 0: 69 ± 25** **Grade 1 or 2 or 3: 47 ± 20** ***p*** **= 0.0043**	**Grade 0: 72 ± 21** **Grade 1 or 2: 53 ± 23** ***p*** **= 0.0127**
8- Sexual functioning	81 ± 30 *p* = 0.80	81 ± 30 *p* = 0.22	**Grade 0: 94 ± 15** **Grade 1: 70 ± 36** **Grade 2 or 3: 47 ± 27** ***p*** **= 0.0003**	**Grade 0: 87 ± 24** **Grade 1 or 2 or 3: 67 ± 36** ***p*** **= 0.0320**	81 ± 30 *p* = 0.19

^a^Student’s *t*-test, ^b^Anova with post hoc pairwise Student’s t-tests

**Table 3:** HAQL Adolescents - Known-groups validity for the proposed score (significant results are given in boldface)

Dimensions	Disease status^a^ HD *N* = 16 ARM *N* = 34	Clinical status			
		Autonomy status ^a^ Usual A. *N* = 37 Lower A. *N* = 9	Soiling ^b^ Grade 0 *N* = 22 Grade 1 *N* = 18 Grade 2 *N* = 3 Grade 3 *N* = 3	Constipation ^b^ Grade 0 *N* = 28 Grade 1 *N* = 3 Grade 2 *N* = 5 Grade 3 *N* = 5	Voluntary bowel movements ^b^ Grade 0 *N* = 25 Grade 1 *N* = 4 Grade 2 *N* = 2 Grade 3 *N* = 3
Overall scale	608 ± 96 *p* = 0.95	**Usual A.: 628 ± 65** **Lower A.: 484 ± 130** ***p*** **= 0.0261**	**Grade 0: 650 ± 39** **Grade 1 or 2 or 3: 564 ± 109** ***p*** **= 0.0224**	**Grade 0 or 1 or 2: 616 ± 85** **Grade 3: 479 ± 137** ***p*** **= 0.0134**	**608 ± 96** ***p*** **= 0.0127**
1- Laxative Diet	89 **±** 18 *p* = 0.73	**Usual A.: 92 ± 16** **Lower A.: 71 ± 21** ***p*** **= 0.0288**	89 **±** 18 *p* = 0.29	**Grade 0: 94 ± 11** **Grade 1 or 2 or 3: 72 ± 23** ***p*** **= 0.0008**	89 **±** 18 *p* = 0.21
2- Presence of Diarrhea	83 **±** 25 *p* = 0.63	84 **±** 25 *p* = 0.18	84 **±** 25 *p* = 0.31	84 **±** 25 *p* = 0.94	84 **±** 25 *p* = 0.44
3- Fecal Continence	86 ± 19 *p* = 0.59	**Usual A.: 90 ± 16** **Lower A.: 62 ± 16** ***P*** **= 0.0008**	**Grade 0: 97 ± 6** **Grade 1: 83 ± 16** **Grade 2: 67 ± 11** **Grade 3: 43 ± 17** ***p*** **< 0.0001**	**Grade 0 or 1: 85 ± 19** **Grade 2 or 3: 69 ± 19** ***p*** **= 0.0067**	**Grade 0: 89 ± 18** **Grade 1 or 2 or 3: 67 ± 13** ***p*** **= 0.0141**
4- Urinary Continence	98 ± 7 *p* = 0.52	98 ± 7 *p* = 0.17	98 ± 7 *p* = 0.47	98 ± 7 *p* = 0.08	98 ± 7 *p* = 0.06
5- Social Functioning	93 ± 20 *p* = 0.55	93 ± 20 *p* = 0.14	93 ± 20 *p* = 0.53	**Grade 0 or 1 or 2: 97 ± 12** **Grade 3: 61 ± 45** ***p*** **= 0.0207**	**93 ± 20** ***P*** **= 0.0009**
6- Emotional Functioning and Body Image	84 ± 21 *p* = 0.84	**Usual A.: 89 ± 14** **Lower A.: 54 ± 24** ***p*** **= 0.0042**	**Grade 0: 96 ± 6** **Grade 1 or 2 or 3: 72 ± 23** ***p*** **= 0.0004**	**Grade 0 or 1 or 2: 87 ± 18** **Grade 3: 53 ± 28** ***p*** **= 0.0020**	**84 ± 21** ***p*** **= 0.0078**
7- Physical symptoms	72 ± 19 *p* = 0.58	72 ± 19 *p* = 0.10	**Grade 0 or 1 or 2: 85 ± 21** **Grade 3: 68 ± 16** ***p*** **= 0.0092**	**Grade 0 or 1 or 2: 73 ± 16** **Grade 3: 48 ± 21** ***p*** **= 0.0129**	72 ± 19 *p* = 0.06

^a^Student’s *t*-test, ^b^ANOVA with post hoc pairwise Student’s t-tests

For adolescents, the overall score could discriminate between different degrees of all clinical status variables. The dimensions “Presence of diarrhea” and “Urinary continence” could not discriminate between any groups. All other dimensions could distinguish between groups of at least one variable. For all clinical status variables there were at least three dimensions that differed significantly per group.

#### Reliability

**Fig. 2:**
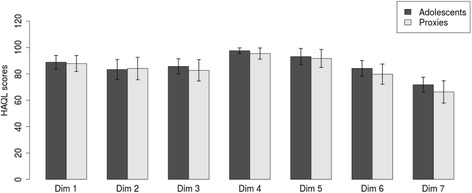
Average outcomes (±2 x standard error) for the dimensions of the proposed HAQL score for adolescents and their proxies

Table 1 presents the reliability results for the proposed structures. Internal reliability was satisfactory for all dimensions, except for “Laxative diet” for the adolescents (α = 0.65). Test-retest concordance was good for all dimensions in both age groups. Responses from adolescents and their proxies correlated strongly and significantly. Their responses did not differ much, although parents typically reported somewhat lower outcomes (Fig. 2).

## Discussion

In this paper a French translation of the Dutch Hirschsprung’s disease Anorectal malformation QoL (HAQL) questionnaire was validated for adults aged 17 years and older and for adolescents, aged between 12 and 16 years. The original structure was found to be unsatisfactory for the French population. It was therefore briefly assessed whether the structure proposed by Clermidi et al. [5] for French children between 6 and 11 was more suitable. It would be a practical advantage if one HAQL questionnaire would suffice in France. However, results were unfavorable. This was expected, since the younger patients are still learning how to cope with their complication [6] and children typically have different priorities than older age groups [13]. Therefore their perception of QoL and their view on their complication may differ from adults, such that one can expect that their QoL should be assessed in a different way.

Finally, a modification of the original structure was proposed, while an effort was made to stay close to the initial structure to optimize international comparability. For adults, the proposed scale consists of 39 items in 8 dimensions and for adolescents it consists of 36 items in 7 dimensions. Dimensions relate to laxative diet, presence of diarrhea, fecal continence, urinary continence, social functioning, emotional functioning, body image and Physical symptoms and sexual functioning (adults only).

The French translation was overall well received by the patients. However, several patients indicated that they did not understand the term “stoma”. For clarity, the description of a stoma in the original questionnaire “A stoma is a derivation of the intestine through the skin, in order to make a short circuit” was changed to “A stoma is a derivation of the intestine through the skin, with a pocket to collect stools” in the new version of the questionnaire.

Questions concerning urinary continence were often considered irrelevant by the recipients. This is understandable, since urinary incontinence occurs only in a minority of HD or ARM patients [1]. However, when it occurs, it is likely to have an impact on a patient’s QoL, such that it is important to keep this dimension in the questionnaire. Questions about sexual functioning had a somewhat higher item nonresponse rate and were reported to be considered irrelevant by a number of patients. Considering the personal nature of such questions, this may be expected. Since the sexual items for adolescents performed badly, these questions were removed from the questionnaire. Finally, for adolescents, results from the patient and proxy questionnaires corresponded well, which suggests that there are no age related problems with understanding the questionnaire.

The statistical results for the proposed HAQL structures were overall satisfactory. Compared to the original structure, the proposed structures contain fewer dimensions with only two items (two instead of four). Since dimensions with less than three items are typically unstable [15], this should result in a more robust structure. Furthermore, reliability results were good, except for dimension “Laxative diet”, where Cronbach’s alpha was just below the threshold (and which is a dimension with only two items). Regarding validity, concurrent validity results were good. However, for adults, dimensions “Laxative diet” and “Urinary continence” were not significantly related to any dimensions of the WHOQOL-BREF questionnaire. Dimension “Laxative diet” has also been reported to perform suboptimal for the Dutch and Italian version of the questionnaire [2, 3], which could be explained by its small number of items. For dimension “Urinary continence” there may be a lack of power to detect significant correlations, since it is a rare functional disorder. The performance of this dimension could improve if a larger sample size would be investigated where there is a better chance of observing this functional disorder.

For adolescents, VSPA dimensions “Relations with family”, “School work” and “Relations with medical staff” did not correlate significantly with any HAQL dimension. This is somewhat surprising, since notably school attitude has been noted to be worse for patients with severe HD or ARM [6].

With respect to known-groups validity, dimension “Urinary continence” could not discriminate between any known groups of patients, which may be due to the aforementioned lack off observed urinary incontinence. The dimension “Laxative diet” did not correlate strongly with any generic QoL dimension for adults. Unexpectedly, dimension “Presence of diarrhea” could not discriminate between known groups for adolescents. Finally, neither the overall score nor any of the dimensions could discriminate between disease statuses. This may simply be due to the small number of HD patients in the sample (*n* = 16 adolescents, *n* = 8 adults), such that the power to detect this group was too low. Alternatively, it might indicate that QoL related differences between HD and ARM patients are small, as also suggested by Hartman et al. [6], although other works question this lack of a difference in QoL [16].

Dimension “Emotional functioning and body image” had the best ability to discriminate between groups. This indicates that patients with varying clinical statuses differ most strongly with regard to emotional functioning and body image. This dimension was also one of the dimensions that was most strongly correlated with the dimensions of the generic QoL questionnaires for both adults and adolescents. This is in line with results from Hartman et al. [17], who identified psychosocial functioning and self-esteem as one of the most important factors affecting the generic QoL of patients with HD and ARM. This highlights the importance of taking psychological consequences for these patients into account.

The French HAQL questionnaire has been validated on patients in France only. Naturally, it would be of interest to use this version in other French speaking countries. It would always be best to statistically validate the questionnaires for each different culture to ensure that they are well understood and interpreted. However, many of the questions in the HAQL questionnaire are directly related to the functioning of the body and are not expected to result in different interpretations across similar cultures (e.g. whether or not a patient lost feces during the night). Therefore, although we recommend a careful consideration of the questions to ascertain that there are no concerns about misinterpretation, the questionnaire may be considered for use in French speaking communities outside of France as well.

## Conclusion

A French version of the Dutch HAQL questionnaire for adults and adolescents is now ready for use in France. It was found that patients with different grades of the Krickenbeck clinical status variables scored differently on the overall HAQL score and its dimensions. This can be especially helpful to enable clinicians to better inform their patients about the physical, social and emotional challenges they might expect.

This newly validated French version of the HAQL questionnaire provides a useful tool for further research on the QoL of HD and ARM patients in France. Such research could aid to answer questions such as whether there are specific subgroups of patients with more severe QoL related problems and what the consequences are of a reduced QoL for HD and ARM patients. Also, potential differences in QoL between HD and ARM patients could be explored further.

The HAQL is now available in the Netherlands, Italy, Sweden and France. A first translation of the HAQL into English has also been made, but needs further validation [14]. To ensure an international QoL assessment tool for HD and ARM patients, it would be highly interesting to continue the translation of the HAQL questionnaire into other languages.

## Additional files

Additional file 1: Tables S1-S8. . (PDF 779 kb)
Additional file 2: The validated questionnaires in the files “HAQL for adolescents”, “HAQL for proxies of adolescents” and “HAQL for adults”. (ZIP 1281 kb)
Additional file 3: The data in the files “data of adolescents” and “data of adults”. (ZIP 5 kb)

## Abbreviations

ARM: Anorectal Malformation
GNEDS: Groupement Nantais d’Ethique dans le Domaine de la Santé
HAQL: Hirschsprung’s disease and Anorectal malformations Quality of Life questionnaire
HD: Hirschsprung’s Disease
ICC: Intraclass Correlation
MTS: Multitrait-Scaling analysis
QoL: Quality of Life
VSPA: Vécu et Santé Perçue de l’enfant et de l’Adolescent questionnaire
WHOQOL-BREF: World Health Organization Quality Of Life questionnaire – abbreviated version
