# Supplementary material for: Validation of the French versions of the Hirschsprung’s disease and Anorectal malformations Quality of Life (HAQL) questionnaires for adolescents and adults
Source: Health Qual Life Outcomes. 2017 Jan 28;15:24. doi: 10.1186/s12955-017-0599-7 (PMC5273813; doi:10.1186/s12955-017-0599-7)
Supplement: Additional file 1: Tables S1-S8. — . (PDF 779 kb) [file 12955_2017_599_MOESM1_ESM.pdf]

Health and Quality of Life Outcomes

Appendix for

Validation of the French versions of the Hirschsprung's disease and Anorectal malformations Quality of Life (HAQL) questionnaires for adolescents and adults.

Corine Baayen[a,b,*], Fanny Feuillet[a,b], Pauline Clermidi[c], Célia Crétolle[d,e], Sabine Sarnacki[d,e], Guillaume Podevin[d,f], Jean-Benoit Hardouin[a]

[a]EA 4275 SPHERE "methodS in Patient-centered outcomes & HEalth ResEarch", University of Nantes, Nantes, France
[b]Plateforme de Biométrie, Département Promotion de la Recherche Clinique, University Hospital of Nantes, Nantes, France
[c]Department of Pediatric surgery, University Hospital of Limoges, France
[d]National French Center of Expertise for Anorectal and Rare Pelvic Malformations, Hospital Necker, Paris, France; University Paris Descartes, Sorbonne Paris Cite, Paris, France; University Hospital of Nantes, Nantes, France
[e]Pediatric Surgery and Urology Department, AP-HP, Hospital Necker, Paris, France; University Paris Descartes, Sorbonne Paris Cite, Paris, France
[f]Department of Pediatric Surgery, University Hospital of Angers, Angers, France

*Corresponding author. Université de Nantes, UFR des Sciences Pharmaceutiques, EA 4275 SPHERE " methodS in Patient-centered outcomes & HEalth ResEarch ", 1 rue Gaston Veil, BP 53508, 44035 Nantes Cedex 1, France. Tel.: +33 2 40 41 29 96; fax: +33 2 40 41 29 96. *E-mail address*: corine.baayen@univ-nantes.fr.

Table A1. HAQL Adults - Multitrait-scaling analysis of original structure. Correlations between items and their corresponding dimension are given in boldface. Items not meeting divergent/convergent validity are given in gray.

	Dim 1	Dim 2	Dim 3	Dim 4	Dim 5	Dim 6	Dim 7	Dim 8	Dim 9	Dim 10
**Dimension 1 - Laxative Diet**										
Item 1	**0.64**	0.15	-0.06	0.08	0.09	0.21	0.30	0.23	0.19	0.13
Item 4	**0.64**	0.02	-0.04	0.04	0.22	0.18	0.22	0.14	0.20	0.11
**Dimension 2 - Constipative Diet**										
Item 2	0.14	**0.44**	0.41	0.41	0.05	0.33	0.37	0.23	0.20	0.15
Item 3	0.02	***0.44***	0.38	*0.53*	0.08	*0.48*	0.43	0.28	0.41	0.22
**Dimension 3 - Presence of Diarrhea**										
Item 29	-0.01	0.47	**0.61**	0.40	0.03	0.23	0.32	0.10	0.30	0.10
Item 30	0.08	0.44	**0.61**	0.46	-0.08	0.47	0.50	0.30	0.38	0.34
**Dimension 4 - Fecal Continence**										
Item 41	-0.03	0.48	0.38	**0.82**	0.06	0.45	0.48	0.43	0.31	0.18
Item 42	0.05	0.39	0.31	**0.75**	-0.08	0.49	0.54	0.37	0.38	0.11
Item 43	-0.02	0.37	0.25	**0.62**	-0.10	0.39	0.48	0.47	0.53	0.50
Item 44	0.12	0.36	0.41	**0.53**	0.06	0.36	0.50	0.49	0.33	0.29
Item 45	-0.07	0.32	0.18	**0.71**	0.36	0.43	0.44	0.45	0.40	0.29
Item 46	0.06	*0.26*	*0.31*	***0.24***	-0.10	0.17	0.14	-0.07	0.16	0.01
Item 47	0.10	0.37	0.24	***0.57***	-0.03	*0.58*	0.43	0.36	0.53	0.24
Item 9	0.13	*0.51*	*0.42*	***0.39***	0.32	*0.56*	*0.60*	*0.44*	*0.40*	0.33
**Dimension 5 - Urinary Continence**										
Item 5	0.09	0.09	-0.05	0.10	**0.85**	0.33	0.25	0.28	0.12	0.14
Item 6	0.09	0.11	0.04	0.20	**0.80**	0.24	0.30	0.39	0.23	0.30
Item 7	0.14	0.05	-0.10	0.05	**0.85**	0.23	0.22	0.29	0.19	0.15
Item 8	0.13	0.08	0.06	0.06	**0.68**	0.30	0.30	0.21	0.23	0.14
**Dimension 6 - Social Functioning**										
Item 49	0.21	0.49	0.20	0.64	0.38	**0.71**	0.64	0.53	0.50	0.41
Item 50	0.12	0.43	0.30	0.56	0.23	**0.86**	0.74	0.67	0.63	0.48
Item 51	0.19	0.42	0.37	0.55	0.26	**0.81**	0.74	0.66	0.55	0.42
**Dimension 7 - Emotional Functioning**										
Item 10	0.28	0.45	0.35	0.49	0.26	0.58	**0.77**	0.65	0.56	0.38
Item 11	0.23	0.26	0.39	0.39	0.32	0.58	**0.71**	0.62	0.53	0.48
Item 14	0.19	0.35	0.24	0.50	0.28	0.62	***0.75***	*0.77*	0.58	0.39
Item 15	0.18	0.45	0.36	0.62	0.29	0.68	**0.83**	0.81	0.57	0.59
Item 16	0.32	0.29	0.16	0.49	0.31	0.56	**0.75**	0.74	0.38	0.48
Item 17	0.12	0.36	0.38	0.54	0.08	0.60	**0.65**	0.58	0.51	0.23
Item 48	0.21	0.53	0.50	*0.72*	0.12	*0.70*	**0.62**	0.53	0.61	0.48
**Dimension 8 - Body Image**										
Item 12	0.07	0.30	0.24	0.60	0.29	0.71	*0.80*	***0.73***	0.50	0.55
Item 13	0.30	0.27	0.15	0.42	0.32	0.56	*0.76*	***0.73***	0.54	0.65
**Dimension 9 - Physical symptoms**										
Item 32	0.29	0.29	0.27	0.33	0.46	0.43	0.39	0.36	**0.63**	0.35
Item 33	0.20	0.14	0.16	0.49	0.31	0.47	0.57	0.46	**0.68**	0.51
Item 34	-0.02	0.20	0.04	0.29	0.00	0.15	*0.35*	*0.34*	***0.32***	0.29
Item 35	0.18	0.11	0.07	0.40	0.44	0.43	0.52	0.49	**0.65**	0.51
Item 36	0.22	0.24	0.30	0.48	-0.04	0.59	*0.68*	0.59	***0.64***	0.52
Item 37	0.05	0.39	0.28	0.40	0.27	0.44	0.43	0.29	**0.60**	0.15
Item 38	0.13	0.37	0.15	0.29	-0.13	0.43	0.28	0.29	**0.52**	0.50
Item 39	0.00	0.34	*0.58*	0.31	-0.11	0.41	0.44	0.27	***0.50***	0.15
Item 40	0.13	0.33	0.50	0.39	0.08	0.38	0.39	0.29	**0.64**	0.33
**Dimension 10 - Sexual functioning**										
Item 18	0.03	0.19	0.21	0.37	0.25	0.49	0.49	0.62	0.52	**0.91**
Item 19	0.19	0.24	0.25	0.36	0.16	0.46	0.56	0.65	0.53	**0.91**

Table A2. HAQL Adolescents - Multitrait-scaling analysis of original structure. Correlations between items and their corresponding dimension are given in boldface. Items not meeting divergent/convergent validity are given in gray.

	Dim 1	Dim 2	Dim 3	Dim 4	Dim 5	Dim 6	Dim 7	Dim 8	Dim 9
**Dimension 1 - Laxative Diet**									
Item 1	**0.48**	0.27	0.37	0.40	0.26	0.42	0.42	0.35	0.41
Item 4	***0.48***	0.19	0.24	0.45	*0.65*	*0.58*	*0.57*	*0.73*	0.46
**Dimension 2 - Constipative Diet**									
Item 2	0.24	***0.49***	*0.74*	0.26	0.21	0.17	0.36	0.39	0.32
Item 3	0.23	***0.49***	*0.73*	0.44	0.41	0.44	*0.55*	0.32	0.28
**Dimension 3 - Presence of Diarrhea**									
Item 26	0.31	*0.82*	***0.77***	0.38	0.34	0.24	0.44	0.24	0.32
Item 27	0.33	0.73	**0.77**	0.27	0.31	0.22	0.46	0.34	0.21
**Dimension 4 - Fecal Continence**									
Item 38	0.24	0.06	0.06	**0.65**	0.25	0.33	0.54	0.37	0.43
Item 39	0.28	0.15	0.10	**0.77**	0.24	0.46	0.65	0.34	0.63
Item 40	0.25	0.35	0.18	***0.54***	0.45	0.39	*0.55*	0.36	*0.66*
Item 41	0.39	0.49	0.33	***0.59***	0.50	*0.68*	*0.66*	0.32	0.45
Item 42	0.32	0.29	0.30	**0.66**	0.30	0.57	0.57	0.31	0.51
Item 43	0.50	0.39	0.20	***0.56***	*0.61*	*0.86*	*0.61*	*0.62*	0.55
Item 44	0.52	0.30	0.26	***0.68***	0.52	*0.70*	0.60	0.44	0.46
Item 9	*0.46*	*0.41*	*0.58*	***0.33***	0.28	*0.42*	*0.58*	*0.41*	0.31
**Dimension 5 - Urinary Continence**									
Item 5	0.40	0.43	0.37	0.48	**0.68**	0.61	0.53	0.59	0.48
Item 6	0.55	0.25	0.18	0.43	**0.83**	0.76	0.62	0.80	0.37
Item 7	0.16	0.22	0.15	0.23	***0.38***	0.37	0.33	*0.42*	0.18
Item 8	0.65	0.22	0.36	0.53	***0.67***	*0.77*	*0.68*	0.61	0.39
**Dimension 6 - Social Functioning**									
Item 46	*0.66*	0.24	0.35	0.59	*0.74*	***0.65***	*0.72*	*0.67*	0.54
Item 47	0.61	0.25	0.17	0.54	*0.78*	***0.64***	*0.69*	*0.82*	0.51
Item 48	0.66	0.14	0.22	0.61	*0.80*	***0.73***	*0.78*	0.73	0.50
Item 17	-0.01	*0.30*	0.02	*0.25*	0.03	***0.21***	*0.25*	0.07	0.07
Item 18	0.03	*0.38*	0.06	*0.52*	0.17	***0.27***	*0.35*	0.08	0.19
**Dimension 7 - Emotional Functioning**									
Item 10	0.62	0.31	0.29	0.60	*0.76*	*0.87*	***0.63***	*0.68*	0.49
Item 11	0.49	0.55	0.44	0.79	0.59	0.69	**0.91**	0.74	0.58
Item 14	0.41	0.41	0.34	0.69	0.57	0.64	**0.71**	0.62	0.56
Item 15	0.54	0.50	0.46	0.68	0.59	0.74	**0.84**	0.76	0.51
Item 16	0.41	*0.66*	*0.51*	0.48	*0.62*	*0.51*	***0.50***	*0.53*	0.31
Item 45	0.40	0.35	0.35	*0.80*	0.29	0.57	***0.66***	0.53	0.51
**Dimension 8 - Body Image**									
Item 12	0.51	0.23	0.13	0.54	*0.84*	*0.77*	*0.68*	***0.62***	0.42
Item 13	*0.63*	0.48	0.38	0.49	0.54	0.54	*0.76*	***0.62***	0.47
**Dimension 9 - Physical symptoms**									
Item 29	0.45	0.01	0.02	0.40	0.15	0.30	0.33	0.29	**0.48**
Item 30	0.32	-0.06	-0.08	0.42	0.28	0.28	0.32	0.25	**0.57**
Item 31	0.26	0.19	0.02	*0.59*	0.28	*0.41*	*0.52*	*0.40*	***0.33***
Item 32	0.62	0.15	0.07	*0.78*	0.43	0.66	*0.68*	0.55	***0.66***
Item 33	0.18	0.26	0.32	0.27	0.08	0.13	0.21	0.09	**0.45**
Item 34	0.24	*0.48*	*0.51*	0.29	0.31	0.15	0.35	0.18	***0.42***
Item 35	0.25	0.15	0.09	0.40	0.30	0.28	0.33	0.24	**0.54**
Item 36	0.21	0.43	0.34	0.38	0.30	0.29	0.36	0.14	**0.44**
Item 37	0.26	0.16	0.21	0.44	0.40	0.31	0.38	0.31	**0.63**

Table A3. Proposed HAQL structures for adults and adolescents

HAQL dimensions	Adults		Adolescents	
	Number of items	Items	Number of items	Items
**Overall scale**	**39**		**36**	
1- Laxative Diet	2	1,4	2	1,4
2- Presence of Diarrhea	4	2,3,29,30	4	2,3,26,27
3- Fecal Continence	6	41,42,43,44,45,47	6	38,39,40,41,42,44
4- Urinary Continence	4	5,6,7,8	4	5,6,7,8
5- Social Functioning	3	49,50,51	3	46,47,48
6- Emotional Functioning and Body Image	10	9,10,11,12,13,14,15,16,17,48	9	9,10,11,12,13,14,15,16,45
7- Physical symptoms	8	32,33,35,36,37,38,39,40	8	29,30,32,33,34,35,36,37
8- Sexual functioning	2	18,19	-	-

Table A4. List of items of the HAQL for adults and adolescents grouped by dimension

HAQL dimensions	Item general meaning	Item number	
		Adults	Adolescents
1- Laxative Diet	Eating special food on purpose to get thin stools	1	1
	Avoid eating special food to maintain thin stools	4	4
2- Presence of Diarrhea	Eating special food on purpose to get thick stools	2	2
	Avoid eating special food to maintain thick stools	3	3
	Thin stools	29	26
	More than four times a day thin stools	30	27
3- Fecal Continence	Loss of feces before reaching the toilet	41	38
	Soiling at daytime	42	39
	Soiling at nighttime	43	40
	Loss of feces at nighttime	44	41
	Loss of feces during physical activity	45	42
	Loss of feces during coughing or sneezing	47	44
4- Urinary Continence	Loss of urine before reaching the toilet	5	5
	Loss of urine during physical activity	6	6
	Loss of urine during emotional moments	7	7
	Loss of urine during coughing or sneezing	8	8
5- Social Functioning	Daily activities	49	46
	Social activities	50	47
	Staying the night elsewhere	51	-
	Play sports	-	48
6- Emotional Functioning and Body Image	Important to be in neighborhood of a toilet	9	9
	Feeling embarrassed	10	-
	Being ashamed of leaving the classroom to go to the toilet	-	10
	Feeling uncertain	15	-
	Feeling worried for the future	11	-
	Feeling that you are teased more than other kids	-	11
	Feeling embarrassed	14	14
	Feeling different	16	15
	Feeling less appreciated by others	17	16
	Feeling afraid that others will smell your feces	48	45
	Feeling less attractive	12	12
	Feeling dissatisfied with your body	13	13
7- Physical symptoms	Feeling swollen (abdomen)	32	29
	Feeling no urge while bowels are full of feces	33	30
	Difficulty to loose feces	35	32
	Difficulty to discriminate between flatulence or feces	36	33
	Being flatulent	37	34
	Difficult to let a flatus	38	35
	Having bowel movements	39	36
	Having abdominal pain	40	37
8 - Sexual functioning	Interest in sex	18	-
	Sexual activity	19	-

Table A5. Concurrent validity: correlations between the dimensions of the HAQL for adults (proposed structure) and the WHOQOL-BREF questionnaire (significant results are given in boldface)

HAQL dimensions	WHOQOL-BREF dimensions			
	**Physical health**	**Psychological health**	**Social relationship**	**Environment**
Overall scale	**0.72**<br>**p<0.0001**	**0.67**<br>**p<0.0001**	**0.56**<br>**p<0.0001**	**0.57**<br>**p<0.0001**
1- Laxative Diet	**0.27**<br>**p=0.0280**	0.11<br>p=0.39	0.21<br>p=0.10	0.14<br>p=0.25
2- Presence of Diarrhea	**0.43**<br>**p=0.0007**	**0.29**<br>**p=0.0340**	**0.28**<br>**p= 0.0395**	**0.34**<br>**p= 0.0108**
3- Fecal Continence	**0.45**<br>**p=0.0005**	**0.31**<br>**p= 0.0217**	**0.32**<br>**p= 0.0192**	**0.27**<br>**p= 0.0415**
4- Urinary Continence	0.10<br>p=0.44	0.23<br>p= 0.07	0.15<br>p= 0.25	0.22<br>p= 0.07
5- Social Functioning	**0.60**<br>**p<0.0001**	**0.58**<br>**p<0.0001**	**0.47**<br>**p= 0.0003**	**0.57**<br>**p<0.0001**
6- Emotional Functioning and Body Image	**0.70**<br>**p<0.0001**	**0.64**<br>**p<0.0001**	**0.50**<br>**p<0.0001**	**0.53**<br>**p<0.0001**
7- Physical symptoms	**0.66**<br>**p<0.0001**	**0.55**<br>**p<0.0001**	**0.47**<br>**p= 0.0004**	**0.61**<br>**p<0.0001**
8- Sexual functioning	**0.49**<br>**P=0.0001**	**0.49**<br>**p= 0.0002**	**0.44**<br>**p= 0.0004**	0.25<br>p=0.05

Table A6. Concurrent validity: correlations between the dimensions of the HAQL for adolescents (proposed structure) and the VSPA questionnaire (significant results are given in boldface)

HAQL dimensions	VSP-Ae dimensions							
	**Relations with family**	**Self esteem**	**Vitality**	**Relations with friends Communication**	**Leisure**	**Psychological & Physical well being**	**School work**	**Relations with medical staff**
Overall scale	0.15<br>p=0.39	**0.70**<br>**p<0.0001**	**0.43**<br>**p=0.0087**	**0.34**<br>**p=0.0409**	**0.55**<br>**p=0.0005**	**0.63**<br>**p<0.0001**	0.14<br>p=0.42	-0.12<br>p=0.51
1- Laxative Diet	-0.05<br>p=0.77	**0.44**<br>**p=0.0033**	**0.35**<br>**p=0.0234**	0.28<br>p=0.08	**0.42**<br>**p=0.0060**	**0.58**<br>**p<0.0001**	0.10<br>p=0.54	-0.03<br>p=0.84
2- Presence of Diarrhea	0.13<br>p=0.43	**0.44**<br>**p=0.0063**	0.26<br>p=0.11	0.11<br>p=0.51	**0.40**<br>**p=0.0155**	0.29<br>p=0.08	-0.02<br>p=0.90	-0.05<br>p=0.77
3- Fecal Continence	0.19<br>p=0.26	**0.41**<br>**p=0.0125**	**0.38**<br>**p=0.0209**	**0.34**<br>**p=0.0404**	**0.34**<br>**p=0.0397**	**0.41**<br>**p=0.0112**	0.29<br>p=0.08	-0.19<br>p=0.30
4- Urinary Continence	0.11<br>p=0.49	**0.46**<br>**p=0.0022**	0.22<br>p=0.17	0.30<br>p=0.06	**0.44**<br>**p=0.0038**	**0.32**<br>**p=0.0354**	-0.11<br>p=0.49	0.13<br>p=0.46
5- Social Functioning	0.07<br>p=0.68	**0.60**<br>**p<0.0001**	0.28<br>p=0.10	0.27<br>p=0.11	**0.39**<br>**p=0.0173**	**0.52**<br>**p=0.0009**	0.16<br>p=0.36	-0.11<br>p=0.56
6- Emotional Functioning and Body Image	0.16<br>p=0.32	**0.75**<br>**p<0.0001**	**0.38**<br>**p=0.0137**	**0.44**<br>**p=0.0036**	**0.56**<br>**p=0.0001**	**0.59**<br>**p<0.0001**	0.23<br>p=0.14	-0.19<br>p=0.25
7- Physical symptoms	0.25<br>p=0.13	**0.46**<br>**p=0.0040**	**0.43**<br>**p=0.0084**	0.32<br>p=0.06	**0.39**<br>**p=0.0156**	**0.59**<br>**p=0.0001**	0.14<br>p=0.42	0.17<br>p=0.34

Table A7. HAQL Adults - Multitrait-scaling analysis of proposed structure. Correlations between items and their corresponding dimension are given in boldface. Items not meeting divergent/convergent validity are given in gray.

	Dim 1	Dim 2	Dim 3	Dim 4	Dim 5	Dim 6	Dim 7	Dim 8
**Dimension 1 - Laxative Diet**								
Item 1	**0.64**	0.14	0.04	0.09	0.21	0.27	0.20	0.13
Item 4	**0.64**	-0.02	0.00	0.22	0.18	0.21	0.21	0.11
**Dimension 2 - Presence of Diarrhea**								
Item 2	0.14	**0.53**	0.35	0.05	0.33	0.36	0.17	0.15
Item 3	0.02	**0.52**	0.45	0.05	0.46	0.43	0.40	0.22
Item 29	0.06	**0.65**	0.35	0.01	0.31	0.31	0.32	0.09
Item 30	0.08	**0.58**	0.37	-0.08	0.47	0.48	0.40	0.34
**Dimension 3 - Fecal Continence**								
Item 41	-0.03	0.51	**0.84**	0.06	0.45	0.49	0.30	0.18
Item 42	0.05	0.43	**0.71**	-0.08	0.49	0.52	0.36	0.11
Item 43	-0.02	0.36	**0.68**	-0.10	0.39	0.48	0.52	0.51
Item 44	0.12	0.44	**0.55**	0.05	0.36	0.50	0.33	0.31
Item 45	-0.07	0.29	**0.74**	0.36	0.43	0.46	0.41	0.29
Item 47	0.10	0.37	**0.58**	-0.03	0.58	0.43	0.52	0.24
**Dimension 4 - Urinary Continence**								
Item 5	0.09	0.00	0.06	**0.85**	0.33	0.29	0.14	0.14
Item 6	0.09	0.08	0.19	**0.80**	0.24	0.34	0.23	0.30
Item 7	0.14	-0.05	0.04	**0.85**	0.23	0.25	0.19	0.15
Item 8	0.13	0.06	-0.01	**0.68**	0.30	0.31	0.25	0.14
**Dimension 5 - Social Functioning**								
Item 49	0.21	0.45	0.58	0.38	**0.71**	0.65	0.51	0.41
Item 50	0.12	0.45	0.50	0.23	**0.86**	0.75	0.64	0.48
Item 51	0.19	0.49	0.49	0.26	**0.81**	0.75	0.57	0.42
**Dimension 6 - Emotional Functioning and Body Image**								
Item 9	0.13	0.56	0.38	0.32	0.56	**0.58**	0.40	0.33
Item 10	0.28	0.45	0.38	0.26	0.58	**0.79**	0.55	0.38
Item 11	0.23	0.36	0.30	0.32	0.58	**0.72**	0.54	0.48
Item 12	0.07	0.30	0.60	0.29	0.71	**0.80**	0.49	0.55
Item 13	0.30	0.22	0.40	0.32	0.56	**0.76**	0.52	0.65
Item 14	0.19	0.28	0.46	0.28	0.62	**0.77**	0.58	0.39
Item 15	0.18	0.47	0.58	0.29	0.68	**0.86**	0.54	0.59
Item 16	0.32	0.25	0.48	0.31	0.56	**0.77**	0.37	0.48
Item 17	0.12	0.42	0.50	0.08	0.60	**0.64**	0.50	0.23
Item 48	0.20	0.60	*0.69*	0.13	*0.69*	***0.62***	0.59	0.48
**Dimension 7 - Physical symptoms**								
Item 32	0.29	0.30	0.30	0.46	0.43	0.40	**0.66**	0.35
Item 33	0.20	0.17	0.48	0.31	0.47	0.56	**0.66**	0.51
Item 35	0.18	0.07	0.41	0.44	0.43	0.51	**0.64**	0.51
Item 36	0.22	0.30	0.45	-0.04	0.59	*0.67*	***0.61***	0.52
Item 37	0.05	0.39	0.36	0.27	0.44	0.42	**0.61**	0.15
Item 38	0.13	0.30	0.26	-0.13	0.43	0.30	**0.52**	0.50
Item 39	0.00	0.49	0.27	-0.11	0.41	0.41	**0.52**	0.15
Item 40	0.13	0.46	0.36	0.08	0.38	0.38	**0.64**	0.33
**Dimension 8 - Sexual functioning**								
Item 18	0.03	0.21	0.35	0.25	0.49	0.54	0.50	**0.91**
Item 19	0.19	0.27	0.34	0.16	0.46	0.59	0.51	**0.91**

Table A8. HAQL Adolescents - Multitrait-scaling analysis of proposed structure. Correlations between items and their corresponding dimension are given in boldface. Items not meeting divergent/convergent validity are given in gray.

	Dim 1	Dim 2	Dim 3	Dim 4	Dim 5	Dim 6	Dim 7
**Dimension 1 - Laxative Diet**							
Item 1	**0.48**	0.36	0.33	0.26	0.44	0.46	0.42
Item 4	***0.48***	0.22	0.38	*0.65*	*0.72*	*0.63*	0.43
**Dimension 2 - Presence of Diarrhea**							
Item 2	0.24	**0.71**	0.19	0.21	0.15	0.42	0.33
Item 3	0.23	**0.71**	0.38	0.41	0.22	0.52	0.25
Item 26	0.31	**0.86**	0.28	0.34	0.21	0.45	0.35
Item 27	0.33	**0.78**	0.18	0.31	0.29	0.48	0.22
**Dimension 3 - Fecal Continence**							
Item 38	0.24	0.06	**0.67**	0.25	0.32	0.52	0.34
Item 39	0.28	0.13	**0.78**	0.24	0.38	0.61	0.55
Item 40	0.25	0.26	***0.58***	0.45	0.35	0.50	*0.62*
Item 41	0.39	0.42	**0.60**	0.50	0.44	0.57	0.41
Item 42	0.32	0.31	**0.65**	0.30	0.48	0.54	0.48
Item 44	0.52	0.29	**0.60**	0.51	0.59	0.60	0.45
**Dimension 4 - Urinary Continence**							
Item 5	0.40	0.45	0.44	**0.68**	0.68	0.54	0.46
Item 6	0.55	0.22	0.39	***0.83***	*0.87*	0.65	0.33
Item 7	0.16	0.18	0.20	**0.38**	0.27	0.36	0.18
Item 8	0.65	0.31	0.47	***0.67***	*0.89*	0.67	0.38
**Dimension 5 - Social Functioning**							
Item 46	0.66	0.31	0.53	0.74	**0.93**	0.73	0.53
Item 47	0.61	0.22	0.50	0.78	**0.85**	0.73	0.47
Item 48	0.66	0.19	0.52	0.80	**0.90**	0.80	0.48
**Dimension 6 - Emotional Functioning**							
Item 9	0.46	0.52	0.33	0.28	0.40	**0.56**	0.31
Item 10	0.62	0.31	0.51	*0.76*	*0.91*	***0.68***	0.47
Item 11	0.49	0.50	0.75	0.59	0.66	**0.88**	0.52
Item 12	0.53	0.20	0.53	*0.83*	*0.84*	***0.69***	0.45
Item 13	0.64	0.43	0.42	0.54	0.60	**0.77**	0.42
Item 14	0.42	0.38	0.68	0.57	0.64	**0.70**	0.53
Item 15	0.55	0.50	0.59	0.59	0.68	**0.88**	0.46
Item 16	0.41	*0.61*	0.43	*0.63*	0.35	***0.53***	0.30
Item 45	0.40	0.35	*0.75*	0.29	0.49	***0.69***	0.43
**Dimension 7 - Physical symptoms**							
Item 29	0.45	0.02	0.40	0.15	0.31	0.35	**0.46**
Item 30	0.33	-0.07	0.43	0.29	0.32	0.31	**0.59**
Item 32	0.62	0.11	*0.76*	0.43	0.60	*0.68*	***0.61***
Item 33	0.18	0.31	0.25	0.08	0.13	0.22	**0.45**
Item 34	0.24	*0.52*	0.22	0.31	0.24	0.34	***0.43***
Item 35	0.25	0.12	0.35	0.30	0.37	0.35	**0.52**
Item 36	0.21	0.40	0.37	0.30	0.21	0.31	**0.48**
Item 37	0.26	0.19	0.46	0.40	0.40	0.36	**0.64**
