# Supplementary material for: Validation of the French versions of the Hirschsprung’s disease and Anorectal malformations Quality of Life (HAQL) questionnaires for adolescents and adults
Source: Health Qual Life Outcomes. 2017 Jan 28;15:24. doi: 10.1186/s12955-017-0599-7 (PMC5273813; doi:10.1186/s12955-017-0599-7)
Supplement: Additional file 2: — The validated questionnaires in the files “HAQL for adolescents”, “HAQL for proxies of adolescents” and “HAQL for adults”. (ZIP 1281 kb) [file 12955_2017_599_MOESM2_ESM.zip › HAQLadultes.pdf]

Questionnaire de qualité de vie

Date : ………/………/………

n° CEMARA : ……………………… Date de naissance : ………/………/…………….

Les questions suivantes concernent votre santé, vos sentiments et votre vie sociale par rapport à votre état de santé.

Toutes les questions concernent les sept jours qui viennent de s'écouler.

Veuillez cocher la réponse qui vous correspond le mieux.

Combien de fois pendant les sept jours précédents :	Jamais	Parfois	Souvent	Très souvent	Ne souhaite pas répondre
1. Avez-vous mangé certains aliments afin de rendre vos selles liquides ?	❒	❒	❒	❒	❒
2. Avez-vous mangé certains aliments afin de durcir vos selles ?	❒	❒	❒	❒	❒
3. Avez-vous évité certains aliments délibérément pour ne pas avoir les selles liquides ?	❒	❒	❒	❒	❒
4. Avez-vous évité certains aliments délibérément pour ne pas avoir les selles dures ?	❒	❒	❒	❒	❒
5. Avez-vous eu des fuites d'urine avant d'arriver aux toilettes ?	❒	❒	❒	❒	❒
6. Avez-vous eu des fuites d'urine pendant une activité physique ? (Par exemple, lorsque vous marchez, vous faites du vélo, vous faites du sport, vous faites l'amour...)	❒	❒	❒	❒	❒
7. Avez-vous eu des fuites d'urine lors des moments d'émotion ? (Par exemple, excitation, anxiété, rire…)	❒	❒	❒	❒	❒
8. Avez –vous eu des fuites d'urine en toussant ou éternuant ?	❒	❒	❒	❒	❒
9. Avez-vous planifié vos activités en fonction de la présence de toilettes à proximité ?	❒	❒	❒	❒	❒

**Combien de fois pendant les sept jours précédents :**	**Jamais**	**Parfois**	**Souvent**	**Très souvent**	**Ne souhaite pas répondre**
10. Vous êtes-vous senti embarrassé à cause de votre état de santé ?	❒	❒	❒	❒	❒
11. Avez-vous été inquièt(e) pour l'avenir à cause de votre état de santé ?	❒	❒	❒	❒	❒
12. Vous sentiez vous moins attirant(e) physiquement à cause de votre état de santé ?	❒	❒	❒	❒	❒
13. Avez-vous été physiquement mal à l'aise ? OU Avez-vous été complexé par votre corps ?	❒	❒	❒	❒	❒
14. Avez-vous eu honte à cause de votre état de santé?	❒	❒	❒	❒	❒
15. Vous sentiez-vous moins sûr de vous à cause de votre état de santé ?	❒	❒	❒	❒	❒
16. Vous sentiez-vous différent des autres à cause de votre état de santé ?	❒	❒	❒	❒	❒
17. Vous sentiez-vous moins apprécié par les autres à cause de votre état de santé ?	❒	❒	❒	❒	❒

<u>Les questions suivantes correspondent à votre vie sexuelle lors des quatre semaines précédentes.</u>

**Combien de fois pendant les quatre semaines précédentes :**	**Jamais**	**Parfois**	**Souvent**	**Très souvent**	**Ne souhaite pas répondre**
18. Avez-vous perdu tout désir sexuel à cause de votre état de santé ?	❒	❒	❒	❒	❒
19. Votre état de santé a-t-il réduit votre activité sexuelle (avec ou sans pénétration) ?	❒	❒	❒	❒	❒

		OUI	NON
20.	Avez-vous une stomie ?	❐	❐

Une stomie est une dérivation de l'intestin vers la peau, avec une poche pour collecter les selles

Non ⇨ Veuillez répondre aux questions **21 à 40**

(Ignorez les questions 41 à 48)

Oui ⇨ Veuillez répondre aux questions **41 à 48**

(Ignorez les questions 21 à 40)

Les questions suivantes (questions 21 à 40) concernent les personnes SANS stomie.

Les questions suivantes concernent la fréquence des certaines choses pendant les sept jours précédents.

**Combien de fois pendant les sept jours précédents :**	**Jamais**	**Parfois**	**Souvent**	**Très souvent**	**Ne souhaite pas répondre**
21. Vos selles étaient liquides ?	❒	❒	❒	❒	❒
22. Avez-vous eu la diarrhée (selles liquides plus de 4 fois par jour) ?	❒	❒	❒	❒	❒
23. Avez-vous été ballonné ?	❒	❒	❒	❒	❒
24. Avez-vous eu des selles dans vos intestins sans avoir envie d'aller aux toilettes ?	❒	❒	❒	❒	❒
25. Avez-vous eu des difficultés à évacuer vos selles ?	❒	❒	❒	❒	❒
26. Avez-vous eu des difficultés à distinguer entre les gaz et les selles ?	❒	❒	❒	❒	❒
27. Avez-vous eu des gaz ?	❒	❒	❒	❒	❒
28. Avez-vous eu des difficultés pour évacuer vos gaz ?	❒	❒	❒	❒	❒

**Combien de fois pendant les sept jours précédents :**	**Jamais**	**Parfois**	**Souvent**	**Très souvent**	**Ne souhaite pas répondre**
29. Avez-vous eu des gargouillements ?	❒	❒	❒	❒	❒
30. Avez-vous eu mal au ventre ?	❒	❒	❒	❒	❒
31. Avez-vous perdu des selles avant d'arriver aux toilettes ?	❒	❒	❒	❒	❒
32. Vos pantalons ont-ils étaient tachés par vos selles, pendant la journée ?	❒	❒	❒	❒	❒
33. Vos pantalons ont-ils étaient tachés par vos selles, pendant la nuit ?	❒	❒	❒	❒	❒
34. Avez-vous perdu des selles pendant votre sommeil ?	❒	❒	❒	❒	❒
35. Avez-vous perdu des selles pendant une activité physique ? (Par exemple, lorsque vous marchez, vous faites du vélo, vous faites du sport, vous faites l'amour...)	❒	❒	❒	❒	❒

**<u>Combien de fois pendant les sept jours précédents :</u>**	**Jamais**	**Parfois**	**Souvent**	**Très souvent**	**Ne souhaite pas répondre**
36. Avez-vous perdu des selles en toussant ou éternuant ?	❒	❒	❒	❒	❒
37. Avez-vous eu peur que l'on puisse sentir vos selles ?	❒	❒	❒	❒	❒
38. Votre état de santé a-t-il réduit vos activités quotidiennes (par exemple le travail, les activités domestiques…) ?	❒	❒	❒	❒	❒
39. Votre état de santé a-t-il réduit vos activités sociales (par exemple, aller voir des amis ou la famille, aller au cinéma ou au restaurant) ?	❒	❒	❒	❒	❒
40. Votre état de santé a-t-il réduit votre envie de passer une nuit quelque part ?	❒	❒	❒	❒	❒

Les questions suivantes (questions 41 à 48) concernent les personnes AYANT une stomie.

Les questions suivantes concernent la fréquence des certaines choses pendant les **sept jours précédents**.

**Combien de fois pendant les sept jours précédents :**	**Jamais**	**Parfois**	**Souvent**	**Très souvent**	**Ne souhaite pas répondre**
41. Vos selles étaient liquides ?	❒	❒	❒	❒	❒
42. Avez-vous eu des fuites au niveau de votre poche de stomie pendant la journée ?	❒	❒	❒	❒	❒
43. Avez-vous eu des fuites au niveau de votre poche de stomie pendant la nuit ?	❒	❒	❒	❒	❒
44. Aviez-vous peur que les gens sentent vos selles ?	❒	❒	❒	❒	❒
45. Aviez-vous peur que les gens voient votre stomie ?	❒	❒	❒	❒	❒
46. Aviez-vous peur que les gens entendent votre stomie ?	❒	❒	❒	❒	❒
47. Avez-vous été inquiété par de possibles fuites au niveau de votre poche de stomie ?	❒	❒	❒	❒	❒
48. Avez-vous eu des difficultés pour vous occuper de votre stomie ?	❒	❒	❒	❒	❒
