# Supplementary material for: Validation of the French versions of the Hirschsprung’s disease and Anorectal malformations Quality of Life (HAQL) questionnaires for adolescents and adults
Source: Health Qual Life Outcomes. 2017 Jan 28;15:24. doi: 10.1186/s12955-017-0599-7 (PMC5273813; doi:10.1186/s12955-017-0599-7)
Supplement: Additional file 2: — The validated questionnaires in the files “HAQL for adolescents”, “HAQL for proxies of adolescents” and “HAQL for adults”. (ZIP 1281 kb) [file 12955_2017_599_MOESM2_ESM.zip › HAQL12to16ansAdolescents.pdf]

Questionnaire de qualité de vie

Date : ………/………/………

n° CEMARA : ………………………   Date de naissance : ………/………/…………….

Les questions suivantes concernent les problèmes dont tu souffres, en relation à ta malformation (maladie de Hirschsprung ou malformation anorectale). Les questions portent également sur la façon dont tu vis ton état de santé.

Les questions ne concernent que les **sept jours qui viennent de s'écouler**. Pour chaque question, tu dois cocher **une seule réponse**. Choisis la réponse qui te correspond le mieux. Il n'y a pas des bonnes et des mauvaises réponses, ce qui importe, **c'est ce que tu penses**.

Essaie de répondre à chaque question.

**Pendant les sept jours précédents :**	**Jamais**	**Parfois**	**Souvent**	**Très souvent**	**Ne souhaite pas répondre**
1. combien de fois as-tu mangé des choses pour que tes selles soient plus liquides ?	❒	❒	❒	❒	❒
2. combien de fois as-tu mangé des choses pour que tes selles soient plus dures ?	❒	❒	❒	❒	❒
3. combien de fois as-tu évité de manger des choses pour ne pas avoir de selles liquides ?	❒	❒	❒	❒	❒
4. combien de fois as-tu évité de manger des choses pour ne pas avoir de selles dures ?	❒	❒	❒	❒	❒
5. combien de fois as-tu eu une fuite urinaire avant d'arriver aux toilettes ?	❒	❒	❒	❒	❒
6. combien de fois as-tu eu une fuite urinaire importante lorsque tu as joué, fait du vélo ou couru ?	❒	❒	❒	❒	❒
7. combien de fois as-tu eu une fuite urinaire importante lorsque tu étais apeuré, en colère, content ou joyeux ?	❒	❒	❒	❒	❒
8. combien de fois as-tu eu une fuite urinaire important lorsque tu as toussé ou éternué ?	❒	❒	❒	❒	❒
9. combien de fois était-il important pour toi d'être près des toilettes ?	❒	❒	❒	❒	❒

**Pendant les sept jours précédents :**	**Jamais**	**Parfois**	**Souvent**	**Très souvent**	**Ne souhaite pas répondre**
10. combien de fois as-tu eu honte car tu devais quitter la salle de cours pour aller aux toilettes ?	❒	❒	❒	❒	❒
11. combien de fois as-tu trouvé que tu étais plus embêté que les autres enfants à cause de ton état de santé ?	❒	❒	❒	❒	❒
12. combien de fois as-tu trouvé que tu étais moins beau que les autres enfants à cause de ton état de santé?	❒	❒	❒	❒	❒
13. combien de fois n'étais-tu pas à l'aise dans ton corps ?	❒	❒	❒	❒	❒
14. combien de fois as-tu été embarrassé à cause de ton état de santé ?	❒	❒	❒	❒	❒
15. combien de fois as-tu trouvé que tu étais différent des autres enfants à cause de ton état de santé ?	❒	❒	❒	❒	❒
16. combien de fois as-tu trouvé que les autres enfants t'aimaient moins à cause de ton état de santé ?	❒	❒	❒	❒	❒

	OUI	NON
17. As-tu une stomie ?	❒	❒

Une stomie est une dérivation de l'intestin vers la peau, avec une poche pour collecter les selles

<u>**Non**</u> ⇨ répond aux questions **18** à **37**.
(Ne répond pas aux questions 38 à 45)

<u>**Oui**</u> ⇨ répond aux questions **38** à **45**.
(Ne répond pas aux questions 18 à 37)

Les questions suivantes (questions 18 à 37) sont seulement pour les enfants SANS stomie.

Essaie de répondre à toutes les questions.

Coches la réponse qui te correspond le mieux.

**Pendant les sept jours précédents :**	**Jamais**	**Parfois**	**Souvent**	**Très souvent**	**Ne souhaite pas répondre**
18. combien de fois tes selles étaient liquides ?	❐	❐	❐	❐	❐
19. combien de fois as-tu fait des selles liquides plus de quatre fois par jour ?	❐	❐	❐	❐	❐
20. combien de fois t'es-tu senti rassasié (ou ballonné) ?	❐	❐	❐	❐	❐
21. combien de fois ne t'es tu pas senti obligé d'aller aux toilettes, alors qu'il y avait des selles dans tes intestins ?	❐	❐	❐	❐	❐
22. combien de fois as-tu eu des difficultés pour évacuer tes selles ?	❐	❐	❐	❐	❐
23. combien de fois as-tu eu des gaz sans le savoir auparavant ?	❐	❐	❐	❐	❐
24. combien de fois as-tu eu des gaz ?	❐	❐	❐	❐	❐
25. combien de fois as-tu eu des difficultés pour avoir des gaz ?	❐	❐	❐	❐	❐

**Pendant les sept jours précédents :**	**Jamais**	**Parfois**	**Souvent**	**Très souvent**	**Ne souhaite pas répondre**
26. combien de fois as-tu eu des gargouillements ?	❐	❐	❐	❐	❐
27. combien de fois as-tu eu mal au ventre ?	❐	❐	❐	❐	❐
28. combien de fois as-tu évacué des selles avant d'arriver aux toilettes ?	❐	❐	❐	❐	❐
29. combien de fois as-tu sali tes sous-vêtements pendant la journée ?	❐	❐	❐	❐	❐
30. combien de fois as-tu sali tes sous-vêtements pendant la nuit ?	❐	❐	❐	❐	❐
31. combien de fois as-tu perdu des selles la nuit ?	❐	❐	❐	❐	❐
32. combien de fois as-tu perdu des selles lorsque tu as joué, as fait du vélo ou as couru ?	❐	❐	❐	❐	❐
33. combien de fois as-tu perdu des selles lorsque tu as toussé ou éternué ?	❐	❐	❐	❐	❐

**Pendant les sept jours précédents :**	**Jamais**	**Parfois**	**Souvent**	**Très souvent**	**Ne souhaite pas répondre**
34. combien de fois as-tu eu peur que tes amis sentent tes selles ?	❒	❒	❒	❒	❒
35. combien de fois es-tu resté chez toi parce que tu avais peur de perdre des selles ?	❒	❒	❒	❒	❒
36. combien de fois as-tu eu envie de jouer à l'extérieur ou chez un ami mais que tu es resté à la maison car tu avais peur de salir tes sous-vêtements ?	❒	❒	❒	❒	❒
37. combien de fois as-tu eu envie de faire du sport mais que tu es resté à la maison car tu avais peur de salir tes sous-vêtements ?	❒	❒	❒	❒	❒

Les questions suivantes (questions 38 à 45) sont seulement pour les enfants AYANT une stomie.

Essaie de répondre à toutes les questions.

Coches la réponse qui te correspond le mieux.

**Pendant les sept jours précédents :**	**Jamais**	**Parfois**	**Souvent**	**Très souvent**	**Ne souhaite pas répondre**
38. combien de fois as-tu eu des selles liquides ?	❒	❒	❒	❒	❒
39. combien de fois y avait-il des fuites au niveau de ta stomie ou de la poche de ta stomie pendant la journée ?	❒	❒	❒	❒	❒
40. combien de fois y avait-il des fuites au niveau de ta stomie ou de la poche de ta stomie pendant la nuit ?	❒	❒	❒	❒	❒
41. combien de fois as-tu eu peur que tes amis sentent tes selles ?	❒	❒	❒	❒	❒
42. combien de fois as-tu eu peur que tes amis arrivent à voir ta stomie ?	❒	❒	❒	❒	❒
43. combien de fois as-tu eu peur que tes amis entendent ta stomie ?	❒	❒	❒	❒	❒
44. combien de fois as-tu eu peur que la poche de ta stomie se mette à fuir ?	❒	❒	❒	❒	❒
45. combien de fois as-tu eu des difficultés pour t'occuper de ta stomie ?	❒	❒	❒	❒	❒
