# Supplementary material for: Validation of the French versions of the Hirschsprung’s disease and Anorectal malformations Quality of Life (HAQL) questionnaires for adolescents and adults
Source: Health Qual Life Outcomes. 2017 Jan 28;15:24. doi: 10.1186/s12955-017-0599-7 (PMC5273813; doi:10.1186/s12955-017-0599-7)
Supplement: Additional file 2: — The validated questionnaires in the files “HAQL for adolescents”, “HAQL for proxies of adolescents” and “HAQL for adults”. (ZIP 1281 kb) [file 12955_2017_599_MOESM2_ESM.zip › HAQL12to16ansProxies.pdf]

Questionnaire de qualité de vie pour les parents

Date : ………/………/………

n° CEMARA : ………………………   Date de naissance : ………/………/……………

Les questions suivantes concernent les problèmes dont votre enfant souffre à cause de son état de santé (malformation ano-rectale ou Maladie de Hirschsprung). Les questions portent également sur la façon dont votre enfant vit son état de santé.

Les questions se focalisent sur les **7 derniers jours**.

Veuillez cocher la réponse qui correspond le mieux à votre enfant.

Pendant les sept derniers jours, combien de fois votre enfant :	Jamais	Parfois	Souvent	Très souvent	Ne sais pas	Ne souhaite pas répondre
1. a-t-il mangé des choses délibérément afin de ramollir ses selles ?	❒	❒	❒	❒	❒	❒
2. a-t-il mangé des choses délibérément afin de durcir ses selles ?	❒	❒	❒	❒	❒	❒
3. a-t-il évité de manger des choses délibérément pour de ne pas avoir de selles liquides ?	❒	❒	❒	❒	❒	❒
4. a-t-il évité de manger des choses délibérément afin de ne pas avoir de selles dures ?	❒	❒	❒	❒	❒	❒
5. a-t-il eu une fuite d'urines avant d'arriver aux toilettes ?	❒	❒	❒	❒	❒	❒
6. a-t-il eu une fuite d'urines importante alors qu'il jouait, faisait du vélo ou courait ?	❒	❒	❒	❒	❒	❒
7. a-t-il eu une fuite d'urines importante lorsqu'il avait peur, était en colère, content ou joyeux ?	❒	❒	❒	❒	❒	❒
8. a-t-il eu une fuite d'urines importante lorsqu'il a toussé ou éternué ?	❒	❒	❒	❒	❒	❒
9. a-t-il été important pour votre enfant d'être près des toilettes ?	❒	❒	❒	❒	❒	❒
10. a-t-il eu honte parce qu'il devait quitter la salle de classe pour aller aux toilettes ?	❒	❒	❒	❒	❒	❒

Pendant les sept derniers jours, combien de fois votre enfant :	Jamais	Parfois	Souvent	Très souvent	Ne sais pas	Ne souhaite pas répondre
11. a-t-il pensé qu'il était plus importuné que les autres enfants à propos de son état de santé ?	❒	❒	❒	❒	❒	❒
12. a-t-il trouvé qu'il était moins beau que les autres enfants à cause de son état de santé ?	❒	❒	❒	❒	❒	❒
13. a-t-il été mécontent de son corps ?	❒	❒	❒	❒	❒	❒
14. a-t-il été gêné de son état de santé ?	❒	❒	❒	❒	❒	❒
15. a-t-il trouvé qu'il était différent des autres enfants à cause de son état de santé ?	❒	❒	❒	❒	❒	❒
16. a-t-il trouvé que les autres enfants l'aimaient moins à cause de son état de santé ?	❒	❒	❒	❒	❒	❒

	OUI	NON
17. Votre enfant a-t-il une stomie ?	❒	❒

Une stomie est une dérivation de l'intestin vers la peau, avec une poche pour collecter les selles

Non ⇨ Veuillez répondre aux questions **18** à **37**

(Ignorez les questions 38 à 45)

Oui ⇨ Veuillez répondre aux questions **38** à **45**

(Ignorez les questions 18 à 37)

Les questions suivantes (questions 18 à 37) sont seulement pour les enfants SANS stomie.

Les questions suivantes concernent la fréquence de certaines choses pendant les 7 derniers jours.

Veuillez cocher la réponse qui correspond le mieux à votre enfant.

**Pendant les sept derniers jours, combien de fois :**	**Jamais**	**Parfois**	**Souvent**	**Très souvent**	**Ne sais pas**	**Ne souhaite pas répondre**
18. les selles de votre enfant ont été liquides ?	❒	❒	❒	❒	❒	❒
19. les selles de votre enfant ont été liquides plus de 4 fois par jour ?	❒	❒	❒	❒	❒	❒
20. votre enfant a-t-il eu l'impression d'avoir le ventre trop plein (ou s'est senti ballonné) ?	❒	❒	❒	❒	❒	❒
21. votre enfant n'a pas senti le besoin d'aller aux toilettes, alors qu'il y avait des selles dans ses intestins?	❒	❒	❒	❒	❒	❒
22. votre enfant a-t-il eu des difficultés pour évacuer ses selles ?	❒	❒	❒	❒	❒	❒
23. votre enfant a-t-il flatulé sans en ressentir le besoin auparavant ?	❒	❒	❒	❒	❒	❒
24. votre enfant a-t-il flatulé ?	❒	❒	❒	❒	❒	❒
25. votre enfant a-t-il eu des difficultés pour flatuler ?	❒	❒	❒	❒	❒	❒

**Pendant les sept derniers jours, combien de fois :**	**Jamais**	**Parfois**	**Souvent**	**Très souvent**	**Ne sais pas**	**Ne souhaite pas répondre**
26. votre enfant a-t-il eu des gargouillements ?	❒	❒	❒	❒	❒	❒
27. votre enfant a-t-il eu mal au ventre ?	❒	❒	❒	❒	❒	❒
28. votre enfant avait-il déjà perdu des selles avant d'arriver aux toilettes ?	❒	❒	❒	❒	❒	❒
29. votre enfant a-t-il sali ses sous-vêtements pendant la journée ?	❒	❒	❒	❒	❒	❒
30. votre enfant a-t-il sali ses sous-vêtements pendant la nuit ?	❒	❒	❒	❒	❒	❒
31. votre enfant a-t-il perdu des selles pendant la nuit ?	❒	❒	❒	❒	❒	❒
32. votre enfant a-t-il perdu des selles lorsqu'il jouait, faisait du vélo ou courait ?	❒	❒	❒	❒	❒	❒

**Pendant les sept derniers jours :**	**Jamais**	**Parfois**	**Souvent**	**Très souvent**	**Ne sais pas**	**Ne souhaite pas répondre**
33. votre enfant a-t-il perdu des selles lorsqu'il a toussé ou éternué ?	❒	❒	❒	❒	❒	❒
34. votre enfant a-t-il eu peur que ses amis puissent sentir ses selles ?	❒	❒	❒	❒	❒	❒
35. votre enfant a-t-il été absent de l'école parce qu'il avait peur de perdre des selles et d'avoir ses sous-vêtements salis par des selles ?	❒	❒	❒	❒	❒	❒
36. votre enfant a-t-il eu envie de jouer à l'extérieur ou chez un ami mais est resté à la maison car il avait peur de salir ses sous-vêtements par des selles?	❒	❒	❒	❒	❒	❒
37. votre enfant a-t-il eu envie de faire du sport mais est resté à la maison car il avait peur de salir ses sous-vêtements par des selles ?	❒	❒	❒	❒	❒	❒

<u>Les questions suivantes (questions 38 à 45) sont seulement pour les enfants AYANT une stomie.</u>

Les questions suivantes concernent la fréquence de certaines choses pendant les 7 derniers jours.

Veuillez cocher la réponse qui correspond le mieux à votre enfant.

**Pendant les sept derniers jours, combien de fois :**	**Jamais**	**Parfois**	**Souvent**	**Très souvent**	**Ne sais pas**	**Ne souhaite pas répondre**
38. votre enfant a-t-il eu des selles liquides ?	❒	❒	❒	❒	❒	❒
39. votre enfant a-t-il eu des fuites de sa stomie ou de la poche de stomie pendant la journée ?	❒	❒	❒	❒	❒	❒
40. votre enfant a-t-il eu des fuites de sa stomie ou de la poche de stomie pendant la nuit ?	❒	❒	❒	❒	❒	❒
41. votre enfant a-t-il eu peur que ses amis puissent sentir ses selles ?	❒	❒	❒	❒	❒	❒
42. votre enfant a-t-il eu peur que des amis puissent voir sa stomie ?	❒	❒	❒	❒	❒	❒
43. votre enfant a-t-il eu peur que des amis puissent entendre sa stomie ?	❒	❒	❒	❒	❒	❒
44. votre enfant a-t-il craint que sa poche de stomie commence à fuir ?	❒	❒	❒	❒	❒	❒
45. votre enfant a-t-il eu des difficultés pour s’occuper de sa stomie ?	❒	❒	❒	❒	❒	❒
